# Knowledge, attitudes, and practice of cervical cancer prevention among health workers in rural health centres of Northern Uganda

**DOI:** 10.1186/s12885-021-07847-z

**Published:** 2021-02-03

**Authors:** James Henry Obol, Sophia Lin, Mark James Obwolo, Reema Harrison, Robyn Richmond

**Affiliations:** 1 University of New South Wales School of Public Health and Community Medicine, Kensington, NSW 2033 Australia; 2 Gulu University, Faculty of Medicine, P. O Box 166, Gulu, Uganda

**Keywords:** Health workers, Cervical cancer, Knowledge, Attitudes, Practice, Northern Uganda, Rural health workers

## Abstract

**Background:**

Cervical cancer is a leading cancer and cause of premature death among women in Uganda aged 15 to 44 years. To address the increasing burden of cervical cancer in Uganda, the Ministry of Health has adopted several strategies which include public education and advocacy. This study aims to assess knowledge, attitudes, and practice of cervical cancer prevention among health workers employed in rural health centres (HCs) III and IV in the Acholi sub-region of Northern Uganda.

**Methods:**

We conducted a cross-sectional survey of nurses, midwives, and clinical officers between February and April 2019 using self-administered questionnaire. We sampled fifty-four HCs III and eight HCs IV. In Uganda, HCs are structured from HC I to HC IV and the health care package provided increases with increasing level of the HC. We used Epidata version 3.1 to create database and analysis was performed using Stata 16. Descriptive and logistic regression analyses were performed. Factors with *p*-values ≤ 0.05 were considered as predictors of outcome.

**Results:**

There were 286 participants who completed the questionnaire: Majority (188, 66%) were females. Nurses were 153 (54%). 141 (75%) female participants self-reported to have been screened for cervical cancer. 171 (60%) participants had adequate knowledge of cervical cancer. 187 (66%) participants had positive attitudes. Participants who indicated not to have ever received training on cervical cancer screening were less likely to have adequate knowledge (AOR = 0.39, 95% CI 0.21–0.71). Participants who indicated not to have ever been trained on cervical cancer screening were less likely to have positive attitudes (AOR = 0.52, 95% CI 0.28–0.97).

**Conclusion:**

Health workers from rural HCs in Uganda play crucial role in cervical cancer prevention as they can reach a wider community. Their significance in the prevention of cervical cancer points to the need for Uganda and other sub-Sahara Africa (SSA) countries to establish training to improve their knowledge, attitudes, and practical skills on cervical cancer screening. Furthermore, Uganda government should develop and disseminate guidelines for cervical cancer prevention to rural health workers to promote standardised cervical cancer prevention activities.

**Supplementary Information:**

The online version contains supplementary material available at 10.1186/s12885-021-07847-z.

## Background

Cervical cancer is the second most common form of cancer and a major cause of premature death among women aged 15 to 44 years worldwide [1]. There is great disparity in the incidence and mortality due to cervical cancer between developed and developing countries [1] with approximately 85% of incidence and 87% of deaths occurring in the less developed regions of the world [2]. Within the African region, Eastern and Southern Africa have the highest incidence and mortality rates of cervical cancer [1]. Furthermore, it has been estimated that from 2013, cervical cancer incidence and mortality will continue to rise in sub-Saharan Africa (SSA) over a period of 20 years [3]. This is in contrast with progress made by Northern Africa which has the lowest incidence and mortality due to cervical cancer in Africa [4]. Therefore, the burden of cervical cancer on the family and society cannot be ignored.

Cervical cancer can be prevented through vaccination with Human Papillomavirus (HPV) vaccine [5, 6]. Furthermore, precancerous cervical lesions remain detectable for up to 10 years or more before progressing to cervical cancer [7]. If precancerous cervical lesions are screened and removed, this can prevent development into cervical cancer [7, 8].

Several challenges create obstacles to the delivery of cervical cancer prevention programmes in SSA, including lack of diagnosis and treatment initiation due to limited service capacity [6, 9, 10] and inadequate supplies and equipment needed for cervical cancer screening and treatment [9, 11]. In addition to health system barriers, studies conducted among healthcare workers from tertiary hospitals in Eastern Africa have identified that most of them lack adequate knowledge of cervical cancer [8, 12, 13], which results in inadequate detection of the disease among women with symptoms of cervical cancer [14, 15].

In Uganda, cervical cancer incidence has been rising at the rate of 1.8% per year over a period of 20 years between 1991 to 2010 [16]. This is due to high prevalence of HPV infection among women (10–40%) [17], high parity [18], early initiation of sex for women [19] and high prevalence of Human immunodeficiency virus/Acquired immunodeficiency syndrome (HIV/AIDS) at 7.5% among women [20]. Despite increasing incidence and high prevalence of risk factors for cervical cancer in Uganda, the proportion of women who have ever been screened is low, estimated to be approximately 5–30% of women [21]. The uptake of the second dose of HPV vaccination for girls 10–14 years is also low at 41% [22] against the target of 80% coverage [7].

To avert the increasing morbidity and mortality associated with cervical cancer globally, World Health Organization (WHO) with its partners have developed the triple-intervention strategy which seeks to ensure that by 2030, 90% of HPV vaccination coverage, 70% of women being screened twice at least in their life time and 90% of women having access to cervical precancerous and cervical cancer treatment and palliative care services [23]. In Uganda, the Ministry of Health developed a Strategic Plan for Cervical Cancer Prevention and Control between 2010 and 2014 [7]. The Strategic Plan sets targets of reaching 90% of Ugandans with health education messages about cervical cancer, screening 80% of women aged 25–49 years for cervical precancerous lesions, and treating those found positive for precancerous cervical lesions [7]. To achieve these targets by the year 2015, the Strategic Plan adopted several implementation strategies which include training health workers employed in HCs levels III and IV on cervical cancer screening using visual inspection with acetic acid (VIA) and treatment of precancerous lesions using cryotherapy [7].

Increased knowledge and positive attitudes of health workers to screen for cervical cancer are critical for cervical cancer prevention. Yet there is no evidence of the level of knowledge and attitudes towards cervical cancer and its prevention among health workers working in rural HCs in Uganda. Adequate knowledge of health workers is a determining factor for positive attitudes, and therefore, it is important that health workers knowledge is periodically assessed with the aim of addressing identified gaps [24]. Health workers are viewed as role models and should provide health education about cervical cancer to the populations they serve [25]. Therefore to achieve the WHO 90–70-90 triple-intervention strategy by 2030 [23, 26] and the goal of the Strategic Plan for Cervical Cancer Prevention and Control in Uganda [7], health workers must have adequate knowledge and positive attitude for them to implement cervical cancer prevention measures.

### Objective

The objective of this study was to assess health workers’ knowledge, attitudes, practice of cervical cancer prevention, and associated factors to help in the development of policy on cervical cancer in Uganda.

## Methods

### Study design

We conducted a cross-sectional survey of health workers working in rural HCs III and IV in Acholi sub-region of Northern Uganda using a self-administered questionnaire which was written in English.

### Setting

The Acholi sub-region in Northern Uganda is bordered by South Sudan on the north, Karamoja on the east, Lango on the south and West Nile on the west. The total land size is around 28,000 km^2^ [27]. Acholi land in Northern Uganda experienced one of the world’s most brutal arm conflicts between 1986 and 2006 [28, 29]. This armed conflict caused massive displacement of 90% of the Acholi population into internally displaced persons camps (IDPs) [28, 30, 31]. The armed conflict disrupted normal healthcare delivery as many health facilities were looted, destroyed or abandoned by the health workers in search of peace or employment elsewhere [32] due to fear of abduction [33]. At the time of the present study, cervical cancer screening and treatment services were provided by St. Mary’s Hospital Lacor, the largest private not-for-profit missionary hospital; Gulu Regional Referral Hospital which is under Uganda Ministry of Health; and non-governmental organizations (NGOs), such as The AIDS Support Organization (TASO), Reproductive Health Uganda and Marie Stopes Uganda. These facilities are located within the urban area of Gulu Municipality.

The study was conducted among health workers who were working in rural HCs III and IV in the eight districts which form Acholi sub-region in Northern Uganda between 11th February to 18th April 2019. At the time of the study, the eight districts had sixty-four HC III and eight HC IV in rural areas [34] (Table 1).

**Table 1:** Numbers of HCs levels III and IV in each of the eight districts

District Name	Number of HC level III	Number of HC level IV
Agago	11	0
Amuru	10	1
Gulu (Aswa county)	6	1
Kitgum	8	1
Lamwo	8	2
Nwoya	3	0
Omoro	7	1
Pader	11	2

### Organization of health facilities in Uganda

The Uganda government operate a decentralised health care delivery system [35, 36]. The primary health care facilities are controlled by the district local government (DLG), and the secondary and tertiary hospitals are semi-autonomous under direct supervision of Ministry of Health [35]. The primary health care facilities comprise of the village health teams (VHTs) which is regarded as a HC I, and then the formal health care system which are HC II – IV and general hospital [35]. The regional referral hospitals are secondary level health facilities while the national referral hospitals are tertiary health facilities [37].

The Uganda Ministry of Health has an established staffing structure for each level of health facility in Uganda [38]. In Uganda, the VHTs are community volunteers chosen by community members to provide healthcare services to their communities [39] and are regarded as HC I [36, 37, 39]. Each VHT serves about 1000 people or 25 households in a village [35, 40, 41], and they provided linkage between the community and the HCs [36, 39, 40, 42]. In addition to other functions, the VHTs are to raise awareness about and mobilise community members to participate in cervical cancer prevention activities [7].

A HC II is the lowest formal health care system [35, 42, 43] and serves a population of about 5000 people [41, 44]. A HC II is managed by an Enrolled Nurse who work with one Enrolled Midwife [37], and provide only outpatient services [35, 42]. The Strategic Plan stipulate that a HC II is to conduct health education, HPV vaccination and referral of women for cervical cancer screening [7].

The HC III is managed by a Senior Clinical Officer and serves a population of around 20,000 people [37, 41]. A Clinical Officer holds a diploma in clinical medicine and community health. Clinical officers carry out diagnosis, treatment as well as general management of patients. They also provide health education and carry out planning as well as drawing budgets for the HCs [45]. According to the Strategic Plan for Cervical Cancer Prevention and Control in Uganda, HC III is expected to perform the following functions: health education and counselling; HPV vaccination of target groups; cervical cancer screening using VIA; and referral of patients to HC IV or hospital for cryotherapy [7]. In HC III, the staff from which our research participants were drawn include 1 Senior Clinical Officer, 1 Clinical Officer, 1 Nursing Officer (Nursing), 2 Enrolled Midwives and 3 Enrolled Nurses [38].

The HC IV serves a population of about 100,000 people [41] and is managed by a senior medical officer [37]. According to the strategic plan, HC IV is expected to perform all the functions performed by HC III and, in addition, treat precancerous lesions using cryotherapy [7]. In HC IV, the staff establishment from which our research participants were also drawn is as follows: 1 Senior Nursing Officer, 1 Nursing Officer (Nursing), 1 Nursing Officer (Midwifery), 3 Enrolled Nurses, 3 Enrolled Midwives and 2 Clinical Officers [38].

The general hospital is the last referral health facility within a district and serves a population of 500,000 people [35, 41]. The general hospital is managed by a Principal Medical Officer and has 1 Medical Officer Special Grade (Obstetrician and Gynaecology), 4 Medical Officers, 1 Principal Nursing Officer, 5 Senior Nursing Officers, 17 Nursing Officer Nursing, 46 Enrolled Nurses, 3 Nursing Officers (Midwifery), 25 Enrolled Midwives, 1 Senior Clinical Officer, and 5 Clinical Officers [37]. A general hospital performs all the functions of a HC IV in addition to treating precancerous lesion using loop electrosurgical excision procedure (LEEP), surgical treatment, referral for cytology, histological diagnosis and staging of cervical cancer [7].

### Health facilities and participants selections criteria

We selected HC III and IV only for the survey because these are the health facilities where rural Ugandan women are to access cervical cancer prevention services [7] since about 72% of Ugandans live in rural areas [46]. The study participants comprised nurses, midwives and clinical officers working in rural HCs III and HCs IV. These participants were selected because the national Strategic Plan for Cervical Cancer Prevention and Control in Uganda specifies the need to train nurses, midwives and clinical officers to provide cervical cancer screening and treatment by 2015 [7]. Each participant must have worked at least for 1 year and has a valid practicing licence and not on study leave.

### Sample size and sampling procedure

The sample size was calculated using the formula for a single population proportion for the survey [47], n = (z^2^pq/d^2^), where n is the desired sample size; z is the 90% confidence interval (CI) which was 1.645; p is the proportion of the health workers with an adequate knowledge, attitude and practice of cervical cancer prevention, set at 0.5 since they were unknown; and d is the level of precision desired, set at 0.05. The total sample size was (1.645)^2^ 0.5(1–0.5)/(0.05)^2^ = 271.

After increasing the calculated sample size to account for a 5% non-response or withdrawal of consent from the study, the final sample size was estimated as 285 health workers. We sampled 54 out of 64 HCs level III based on the Ministry of Health national health facility master list 2018 [34]. The 54 HCs III were selected as follows: All HC III names were listed alphabetically and assigned numbers from 1 to 64. We generated 54 random numbers between 1 and 64 using software [48] and these numbers were matched with the one for the HCs giving us the names of HCs for the survey. The study was advertised in each of the HCs and potential research participants were briefed about the study. Consenting research participants were consecutively recruited from each of the sampled HCs.

### Operational definitions and measurements

**Knowledge:** Knowledge was evaluated using 25 question items with a “yes” or “no” response. Each correct response was awarded one mark and a wrong response given zero marks. There were 9 questions on cervical cancer risk factors; 5 on cervical cancer signs and symptoms; and 11 questions on cervical cancer prevention and control. The scores from all the 25 items were summed and the mean sum of total scores was calculated. Any participant who scored equal or above the mean (≥18 marks) was categorised as having adequate level of cervical cancer knowledge and participants who scored below the mean (less than 18 marks) were categorised as having inadequate knowledge for cervical cancer.

#### Attitudes

We assessed attitudes using 13 statement items measured on 5-point Likert scale (1 strongly disagree, 2 disagree, 3 neutral, 4 agree and 5 strongly agree) to measure proxy variables of willingness to participate in cervical cancer training, screening, and other prevention activities. To sum the scores, individual responses from statements which were formulated negatively were transformed to positive statements. The participants’ scores from all the 13 statements were summed-up and the average score was calculated. Participants who scored equal or above the mean (≥56) were considered as having positive attitudes while participants who scored below the mean were considered as having negative attitudes.

The scoring methods were adopted as there are no standardised test questions with cut-off values for participants’ knowledge and attitudes in this region. However, these scores reflect participants’ knowledge and attitudes towards participation in cervical cancer training, and prevention activities.

### Data sources and measurements

Data was collected using a self-administered structured questionnaire (see Additional file 1) which was written in English. The questions for the questionnaire were selected based on the review of relevant literature which address the study objectives. These includes the Uganda Strategic Plan for the Prevention and Control of Cervical Cancer [7] and other studies conducted within Uganda, SSA and elsewhere [8, 13, 18, 49–58]. The questionnaire consisted of five parts. The first part asked about demographic factors including age, gender, qualifications, HC level, whether HC staff received training to conduct cervical cancer screening or not, and the number of years the participant had worked. We also obtained data on HC characteristics; namely, availability of guideline for HPV vaccination and cervical cancer screening; whether HC received funding for cervical cancer activities and health education material; and whether health workers conducted outreach health education in the community. The second section sought information on when they had heard of cervical cancer for the first-time and the sources of information. The third part of the questionnaire obtained information about cervical cancer screening uptake by female health workers and the reasons for not undertaking cervical cancer screening. For male health workers, we asked whether they would encourage their partners to get screened for cervical cancer. Part four of the questionnaire contained questions about knowledge on cervical cancer risk factors, signs, symptoms, and methods of cervical cancer prevention. We included questions about cervical cancer myths to test the health workers. Questions in part five asked information about participants’ attitudes towards participation in cervical cancer training, and prevention activities.

### Quality control

The following quality control procedures were taken to minimise bias: The principal investigator (PI) trained the research assistants on data collection tool, consent procedure, confidentiality, and privacy. The PI together with research assistants pre-tested the questionnaire among 15 health workers (nurses, midwives, and clinical officers) working in three HCs III in Gulu Municipality. The information collected during pre-testing was used to validate the content and to modify the questionnaire to improve clarity and ease of understanding before using the questionnaire in the study. The information obtained from the 15 health workers during pre-testing the questionnaire was excluded in the final analysis reported in this manuscript. The PI and research assistant closely monitored filling in the questionnaires to ensure that the questionnaires were correctly filled, and participants do not discuss answers among themselves or search information online.

### Data management and analysis

The PI entered the data into Epidata 3.1 [59] database and analysed using Stata version 16 [60]. Participants ages were categorised using interval of 5 years while participants’ number of years of working as a health worker was categorised using interval of 4 years. Knowledge and attitudes were categorised as described under the operational definitions of outcomes measures. One participant did not provide answer for knowledge or attitude and was omitted in the analysis of knowledge and attitude. Categorical variables were summarised and displayed in tables with frequencies and percentages. Chi square test and Fisher’s exact test were used to compare proportions among health worker qualifications and score for adequate knowledge of cervical cancer, cervical cancer risk factors, signs and symptoms, and prevention methods. Chi square test and Fisher’s exact test were used to compare proportions among health worker qualifications and average score for attitudes. Bivariate analyses were performed to examine the relationship between dependent and independent variables using logistic regression analysis. Determinant of knowledge and attitudes were investigated using multivariate logistics regression analysis using enter methods. Any variable with a *p*-value ≤ 0.05 was considered a statistically significant predictor of adequate knowledge and positive attitudes.

## Results

### Study population

A total of 301 health workers were contacted for participation in the study. Ten health workers declined to participate in the study expressing disinterest. Five participants did not return their questionnaire when they were called to a medical emergency or delivery and we made effort to retrieve the questionnaires, but we were unsuccessful. These participants were regard to have withdrawn their consent for further participation in the study. This leaves us with 286 health workers who filled and handed back the questionnaire.

### Demographic characteristics of respondents

A total of 286 participants aged 21–57 years were surveyed with a mean age of 35 years. Just over half of the participants were nurses (*n* = 153, 54%). Most participants were female (*n* = 188, 66%). The average years of work experience was 9.8 (range 1–38 years) with most participants having between 5 and 9 years’ experience (*n* = 113, 40%). Most of the participants were from HC III (*n* = 222, 78%). A third of the participants were ever trained to conduct cervical cancer screening (*n* = 93, 33%). Less than half of the participants were conducting outreach health education about cervical cancer in the community (*n* = 128,45%). Health workers who indicated that their HC had HPV vaccination guideline were 205 (74%). Health workers who indicated that their HCs had guideline for cervical cancer screenings were 58 (21%). Table 2 summarises the demographic characteristics of the study participants.

**Table 2:** Demographic characteristics of the health workers

Variables	Frequency	Percent
**Health workers qualifications (*n*** **= 286)**		
Nurses	153	54
Midwife	81	28
Clinical Officer	52	18
**Gender of health workers (*n*** **= 286)**		
Male	98	34
Female	188	66
**Health centre level of health workers (*n*** **= 286)**		
Health centre level IV	64	22
Health centre level III	222	78
**Number of years employed as a health worker (*n*** **= 281)**		
1–4 years	54	19
5–9 years	113	40
10–14 years	53	19
15 + years	61	22
**Age group of health workers (*n*** **= 280)**		
21–25 years	19	7
26–30 years	76	27
31–35 years	72	26
36–40 years	52	18
41+ years	61	22
**Health worker trained to conduct cervical cancer screening (*n*** **= 283)**		
Yes	93	33
No	190	67
**HC has education material about cervical cancer (*n*** **= 284)**		
No	200	70
Yes	84	30
**Health worker conducts outreach health education in the community (*n*** **= 283)**		
Yes	128	45
No	155	55
**HC receives fund for cervical cancer activities (*n*** **= 282)**		
No	273	97
Yes	9	3
**HC has guideline for cervical cancer screening (*n*** **= 281)**		
Yes	58	21
No	223	79
**HC has HPV vaccination guideline (*n*** **= 276)**		
Yes	205	74
No	71	26
**Participant knew period covered by strategic plan for cervical cancer prevention and control in Uganda**		
Yes	5	2
No	281	98

*HC* Health centre, *HPV* Human Papillomavirus

### Practice of cervical cancer prevention

**Fig. 1:**
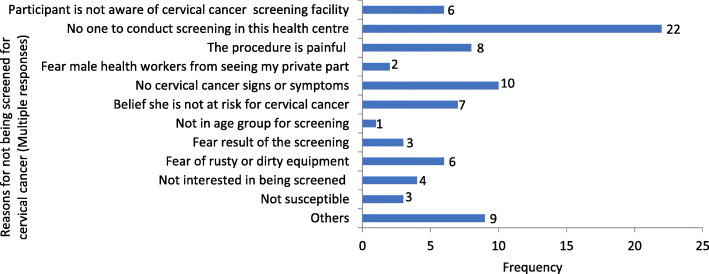
Reasons for not undertaking cervical cancer screening by 47 female health workers

There were 141 (75%) female health workers who self-reported to have ever been screened for cervical cancer, while 87 (91%) of the male health workers indicated they had encouraged their partners to be screened for cervical cancer. Figure 1 summarises reasons for not undertaking cervical cancer screening by 47 female health workers.

### Knowledge about cervical cancer

Participants had heard about cervical cancer from these sources: through the media 98 (35%), during training for current job 146 (51%), from colleagues at work 11 (4%), during in-service training 23 (8%) and other sources 6 (2%). We found that 171 (60%) participants had adequate knowledge i.e., they scored equal to or above mean (≥18) out of a total of 25. Overall score for adequate knowledge about cervical cancer among nurses, midwives and clinical officers was not significant h (Χ^2^ = 0.16, *p*-value = 0.93). However, there were associations among the following knowledge variables and health workers’ qualification: tobacco smoking and risk factor for cervical cancer (Χ^2^ = 8.37, *p*-value = 0.02); unexplained weight loss as sign for cervical cancer (Χ^2^ = 6.46, *p*-value = 0.04); avoiding multiple deliveries/births as method for cervical cancer prevention (Χ^2^ = 10.11, *p*-value = 0.01); and knowing correct age group for screening using VIA according to Uganda Ministry of Health (Χ^2^ = 6.23, *p*-value = 0.04).

Nearly all participants (*n* = 283, 99%) correctly identified having multiple sexual partners as a risk factor for cervical cancer. Tobacco smoking as a risk factor for cervical cancer was indicated by 120 (42%). Long term use of oral contraceptives as a risk factor for cervical cancer was identified by 65 (23%). Infection with Chlamydia *trachomatis* as a risk factor for cervical cancer was identified by 142 (50%). Most participants (*n* = 266, 93%) indicated that bleeding after sexual intercourse is a sign of cervical cancer. Participants knew about prevention of cervical cancer such as: avoiding prolonged use of oral contraceptives pills (*n* = 81, 28%) and abstaining from tobacco use (*n* = 133, 47%). There were 277 (97%) participants who knew that early screening is a preventive measure, but only 46 (16%) knew the correct age group for cervical cancer screening using VIA based on the Uganda Ministry of Health guideline (Table 3).

**Table 3:** Knowledge about cervical cancer risk factors, signs and symptoms, and prevention and control stratified by health worker’s qualifications

Variables	Total, ***N*** = 285 (%)	Nurses, ***N*** = 153 (53%)	Midwives, ***N*** = 80 (28%)	CO, ***N*** = 52 (18%)	χ2	***p***-value
Adequate knowledge score ≥ mean (18 marks)	171 (60)	93 (61)	48 (60)	30 (58)	0.16	0.93
**Risk factors for cervical cancer**						
Infection with HPV	244 (85)	130 (85)	66 (82.5)	48 (92)	2.57	0.28
**Smoking cigarette**	**120 (42)**	**54 (35)**	**36 (45)**	**30 (58)**	**8.37**	**0.02**
Infection with HIV	235 (82)	124 (81)	66 (82.5)	45 (87)	0.81	0.69
Having multiple deliveries	189 (66)	110 (72)	50 (62.5)	29 (56)	5.24	0.07
Early age at first delivery	177 (62)	92 (60)	55 (69)	30 (58)	2.18	0.35
Early age at sexual debut	264 (92)	139 (91)	74 (93	51 (98)	2.97	0.22
Long-term use of oral contraceptives pills	65 (23)	35 (23)	21 (26)	9 (17)	1.43	0.49
Infection with Chlamydia trachomatis	142 (50)	83 (54)	35 (44)	24 (46)	2.66	0.26
Having multiple sexual partners	283 (99)	153 (100)	78 (98)	52 (100)	5.16	0.08
**Signs and symptoms of cervical cancer**						
Foul-smelling vaginal discharge	237 (83)	124 (81)	69 (86)	44 (85)	1.11	0.58
Bleeding after menopause	260 (91)	138 (90)	73 (91)	49 (94)	0.79	0.70
Bleeding after sexual intercourse	266 (93)	143 (93)	73 (91)	50 (96)	1.22	0.54
Abnormal vaginal bleeding between period	237 (83)	126 (82)	67 (84)	44 (85)	0.17	0.92
**Unexplained weight loss**	**174 (61)**	**95 (62)**	**41 (51)**	**38 (73)**	**6.46**	**0.04**
**Prevention and control of cervical cancer**						
Vaccination against HPV	282 (99)	152 (99)	78 (97.5)	52 (100)	2.40	0.30
Correct age group for HPV vaccination according to Uganda Ministry of Health	250 (87)	138 (90)	68 (84)	44 (85)	2.33	0.31
Using condom during sexual encounter	234 (82)	126 (82)	66 (82.5)	42 (81)	0.08	0.96
Avoiding multiple sexual partners	280 (98)	149 (97)	79 (98)	52 (100)	1.37	0.74
Delaying initiation of sex after 18 years	220 (77)	114 (75)	62 (77.5)	44 (85)	2.26	0.32
Male circumcision	249 (87)	134 (88)	68 (85)	47 (90)	0.84	0.66
Avoiding prolong use of oral contraceptive pills	81 (28)	41 (27)	28 (35)	12 (23)	2.63	0.27
Avoiding smoking cigarette	133 (47)	64 (42)	40 (50)	29 (56)	3.53	0.17
**Avoiding multiple deliveries/births**	**193 (68)**	**113 (74)**	**54 (67.5)**	**26 (50)**	**10.11**	**0.01**
Early screening	277 (97)	148 (97)	78 (97.5)	51 (98)	0.30	0.86
**Correct age group for screening using VIA according to Uganda Ministry of Health**	**46 (16)**	**19 (12)**	**20 (25)**	**7 (13)**	**6.23**	**0.04**

χ2 **=** chi-square test, df = 2, Significant results are bolded, *CO* Clinical Officers

### Misconceptions about cervical cancer risk factors, signs, and symptoms

Participants had misconceptions about cervical cancer risk factors. Most participants believed that family history was a risk factor for cervical cancer (*n* = 209, 73%); half (*n* = 144, 50%) believed cervical cancer was caused by infection with Herpes simplex virus. Few believed excessive alcohol consumption was a risk factor (*n* = 48, 17%). There were also some misconceptions regarding the signs and symptoms of cervical cancer. Two thirds believed abdominal pain was a sign of cervical cancer (*n* = 192, 67%); 35 participants (12%) thought headaches were a symptom, and 18 (6%) thought night sweats were also associated (Table 4).

**Table 4:** Misconception about cervical cancer risk factors, signs and symptoms stratified by health workers’ qualifications

Variables	Total ***N*** = 285 (%)	Nurses, ***N*** = 153 (53%)	Midwives, ***N*** = 80 (28%)	CO, ***N*** = 52 (18%)	χ2	***P***-value
**Misconception about cervical cancer risk factors**						
Infection with herpes simplex virus-2 (HSV-2)	144 (51)	75 (49)	40 (50)	29 (56)	0.72	0.70
Over consumption of Alcohol	48 (17)	23 (15)	14 (17.5)	11 (21)	1.07	0.59
Having a family history of cervical cancer	209 (73)	120 (78)	56 (70)	33 (63)	5.08	0.08
**Misconception about cervical cancer signs and symptoms**						
Abdominal pain	192 (67)	101 (66)	52 (65)	39 (75)	1.71	0.43
Having headache	35 (12)	24 (16)	6 (7.5)	5 (10)	3.69	0.16
Having a lot of night sweat	18 (6)	7 (5)	7 (9)	4 (8)	1.75	0.42

χ2 **=** chi-square test, df = 2, *CO* Clinical Officers

### Attitudes towards participation in cervical cancer training, and prevention activities

There were 187 (66%) health workers who had positive attitudes i.e., they scored equal or above mean (≥56 out of 65). There was no association between attitudes and health workers qualification (Χ^2^ = 3.16, *p*-value = 0.206). A total of 250 (88%) participants strongly agreed that they would be happy to see that their daughters or sisters were immunised against HPV. Participants who strongly agreed that they would participate in training for cervical cancer prevention if organised by Uganda Ministry of Health were 199 (70%). There were 256 (90%) who strongly disagreed with the statement that cervical cancer is not a serious health problem, so screening is just a burden. There were 214 (75%) participants who strongly agreed that they were more likely to screen women for cervical cancer if they were trained and provided with equipment and consumables. Table 5 summarises participant’s responses to the various statements about participation in cervical cancer training, and prevention activities.

**Table 5:** Participants’ attitudes towards participation in cervical cancer training, and prevention activities in Northern Uganda

Statements	N	Strongly Disagree (1)	Disagree (2)	Neutral (3)	Agree (4)	Strongly Agree (5)
I always advise my patients to screen for cervical cancer	283	10 (4)	2 (1)	21 (7)	97 (34)	153 (54)
I discuss cervical cancer in our staff meeting	279	39 (14)	73 (26)	49 (18)	87 (31)	31 (11)
I will be happy to see that my children/sister are immunised against HPV	284	8 (3)	0 (0)	2 (1)	24 (8)	250 (88)
Cervical cancer is not a serious health problem, so screening is just a burden	285	256 (90)	18 (6)	2 (1)	3 (1)	6 (2)
Even if we screen and find a woman with precancerous lesion, there is nothing we can do	280	198 (71)	61 (22)	10 (3)	3 (1)	8 (3)
I do not think it is necessary to screen for cervical cancer in our health facility	285	236 (83)	30 (10)	0 (0)	3 (1)	16 (6)
Government has not shown commitment about cervical cancer so why bother us	284	159 (56)	69 (24)	23 (8)	17 (6)	16 (6)
I am not interested in cervical cancer prevention because partners/NGOs do their work without developing our capacity to implement cervical cancer control program on our own	284	131 (46)	81 (28)	28 (10)	28 (10)	16 (6)
I will participate in cervical cancer prevention program if I am going to be paid money cash	282	90 (32)	102 (36)	49 (17)	19 (7)	22 (8)
I am willing to participate in a training for cervical cancer prevention if organised by Gulu University & University of New South Wales – Australia	283	8 (3)	8 (3)	15 (5)	61 (22)	191 (67)
I am willing to participate in a training for cervical cancer prevention if organised by Ministry of Health Uganda	283	4 (1)	3 (1)	11 (4)	66 (23)	199 (70)
I am willing to participate in a training for cervical cancer prevention if organised by NGOs	282	5 (2)	6 (2)	20 (7)	77 (27)	174 (62)
I am more likely to screen women for cervical cancer if I am trained and given equipment & consumables	284	8 (3)	1 (0)	2 (1)	59 (21)	214 (75)

### Factors associated with adequate knowledge about cervical cancer among health workers

In the multivariate logistic regression model, participants who indicated not to have ever received training to conduct cervical cancer screening were 61% less likely to have an adequate knowledge score (AOR = 0.39, 95% CI 0.21–0.72, *p*-value = 0.00) (Table 6).

**Table 6:** Bivariate and multivariate analysis of factors associated with adequate knowledge scores about cervical cancer among health workers

Demographic and HC characteristics	n (%)	Knowledge		COR	95% CI	***P***-value	AOR	95% CI	***P***-value
		Inadequate ***n*** = 114 (40)	Adequate ***n*** = 171 (60)						
**Health workers qualifications (*n*** **= 285)**									
Nurses	153 (54)	60 (39)	93 (61)	1			1		
Midwife	80 (28)	32 (40)	48 (60)	0.97	0.56–1.68	0.91	1.15	0.57–2.30	0.70
Clinical Officer	52 (18)	22 (42)	30 (58)	0.88	0.46–1.67	0.69	0.98	0.45–2.15	0.97
**Gender of health workers (*n*** **= 286)**									
Male	98 (34)	42 (43)	56 (57)	1			1		
Female	187 (66)	72 (39)	115 (61)	1.20	0.73–1.97	0.48	1.43	0.74–2.76	0.28
**Health centre level of health workers (*n*** **= 286)**									
Health centre level IV	64 (22)	26 (41)	38 (59)	1			1		
Health centre level III	221 (78)	88 (40)	133 (60)	1.03	0.59–1.82	0.91	1.10	0.56–2.16	0.78
**Numbers of years employed as a health worker (*n*** **= 281)**									
1–4 years	54 (19)	27 (50)	27 (50)	1			1		
5–9 years	112 (40)	42 (37.5)	70 (62.5)	1.67	0.86–3.21	0.13	2.07	0 .84–5.11	0.11
10–14 years	53 (19)	20 (38)	33 (62)	1.65	0.76–3.56	0.20	1.17	0.38–3.62	0.79
15 + years	61 (22)	24 (39)	37 (61)	1.54	0.74–3.23	0.25	0.91	0.22–3.65	0.89
**Age group of health workers (*n*** **= 280**									
21–25 years	19 (7)	9 (47)	10 (53)	1			1		
26–30 years	76 (27)	35 (46)	41 (54)	1.05	0.39–2.89	0.92	0.83	0.24–2.88	0.77
31–35 years	71 (25)	27 (38)	44 (62)	1.47	0.53–4.07	0.46	0.87	0.21–3.57	0.85
36–40 years	52 (19)	20 (38)	32 (62)	1.44	0.50–4.16	0.50	1.32	0.30–5.72	0.71
41+ years	61 (22)	23 (38)	38 (62)	1.49	0.53–4.20	0.45	1.64	0.29–9.31	0.58
**Healthcare worker trained to conduct cervical cancer screening (*n*** **= 282)**									
Yes	93 (33)	26 (28)	67 (72)	1			1		
No	189 (67)	85 (45)	104 (55)	0.47	0.28–0.81	0.01	**0.39**	**0.21–0.72**	**< 0.001**
**HC has education material about cervical cancer (*n*** **= 283)**									
Yes	84 (30)	33 (39)	51 (61)	1			1		
No	199 (70)	80 (40)	119 (60)	0.96	0.57–1.62	0.89	1.17	0.62–2.23	0.63
**Health worker conduct outreach health education in the community (*n*** **= 283)**									
Yes	128 (45)	45 (35)	83 (65)	1			1		
No	154 (55)	69 (45)	85 (55)	0.67	0.41–1.08	0.10	0.73	0.41–1.30	0.28
**HC has guideline for cervical cancer screening (*n*** **= 281)**									
Yes	58 (21)	18 (31)	40 (69)	1			1		
No	222 (79)	94 (42)	128 (58)	0.61	0.33–1.13	0.12	0.53	0.25–1.12	0.10
**HC has HPV vaccination guideline (*n*** **= 275)**									
Yes	204 (74)	76 (37)	128 (63)	1			1		
No	71 (26)	33 (46)	38 (54)	0.68	0.40–1.18	0.17	0.68	0.38–1.25	0.21

*COR* Crude Odds Ratio, *AOR* Adjusted Odds Ratio. Adjusted for each demographic characteristic. Bolded figures indicate statistically significant variables at *p* ≤ 0.05. *HC* Health centre. We used Hosmer-Lemeshow goodness-of-fit test to check if our final model had fitted the data perfectly well and the *p*-value = 0.44 from the Hosmer-Lemeshow’s goodness-of-fit test is an indicator that our model fitted the data well. The few missing data on demographic characteristics were not dropped from the analysis since the missing rates were less than 5% and were considered inconsequential based on Schafer (1999)

### Factors associated with positive attitudes towards participation in cervical cancer training, and prevention activities

Participants who indicated not to have ever been trained to conduct cervical cancer screening were 48% time less likely to have positive attitudes towards participation in cervical cancer training, and prevention activities (AOR = 0.52, 95% CI: 0.28–0.97, *p*-value = 0.04) (Table 7).

**Table 7:** Bivariate and multivariate analysis of demographic characteristics associated with positive attitudes towards participation in cervical cancer training, and prevention activities by health workers (*n* = 285)

Demographic characteristics	n (%)	Attitudes		COR	95% CI	***p***-values	AOR	95% CI	***p***-value
		Negative ***n*** = 98 (34%)	Positive ***n*** = 187 (66%)						
**Health workers qualifications (*n*** **= 285)**									
Nurses	153 (54)	47 (31)	106 (69)	1			1		
Midwife	80 (28)	28 (35)	52 (65)	0.82	0.46–1.46	0.51	0.56	0.28–1.16	0.12
Clinical Officer	52 (18)	23 (44)	29 (56)	0.56	0.29–1.07	0.08	0.55	0.25–1.22	0.14
**Gender of health workers (*n*** **= 285)**									
Male	98 (34)	37 (38)	61 (68)	1			1		
Female	187 (66)	61 (33)	126 (67)	1.25	0.75–2.09	0.39	1.17	0.60–2.30	0.64
**Health centre level of health workers (*n*** **= 285)**									
Health centre level IV	64 (22)	24 (37.5)	40 (62.5)	1			1		
Health centre level III	221 (78)	74 (33)	147 (67)	1.19	0.67–2.12	0.55	1.25	0.65–2.43	0.51
**Numbers of years employed as a health worker (*n*** **= 280)**									
1–4 years	54 (19)	19 (35)	35 (65)	1			1		
5–9 years	112 (40)	43 (38)	69 (62)	0.87	0.44–1.71	0.69	1.1	0.44–2.72	0.84
10–14 years	53 (19)	13 (25)	40 (75)	1.67	0.72–3.86	0.23	3.17	0.96–10.54	0.06
15 + years	61 (22)	22 (36)	39 (64)	0.96	0.45–2.07	0.92	2.91	0.68–12.47	0.15
**Age group of health workers (*n*** **= 279)**									
21–25 years	19 (7)	6 (32)	13 (68)	1			1		
26–30 years	76 (27)	26 (34)	50 (66)	0.89	0.30–2.61	0.83	1.02	0.28–3.67	0.98
31–35 years	71 (25)	26 (37)	45 (63)	0.8	0.27–2.35	0.68	0.65	0.15–2.76	0.56
36–40 years	52 (19)	12 (23)	40 (77)	1.54	0.48–4.92	0.47	0.92	0.20–4.14	0.91
41+ years	61 (22)	25 (41)	36 (59)	0.66	0.22–1.98	0.46	0.27	0.05–1.58	0.15
**Healthcare worker trained to conduct cervical cancer screening (*n*** **= 283)**									
Yes	93 (33)	25 (27)	68 (73)	1			1		
No	190 (67)	72 (38)	117 (62)	0.6	0.35–1.03	0.06	**0.52**	**0.28–0.97**	**0.04**
**HC has education material about cervical cancer (*n*** **= 283)**									
Yes	84 (30)	24 (29)	60 (79)	1			1		
No	200 (70)	74 (37)	125 (63)	0.68	0.39–1.18	0.17	0.7	0.36–1.34	0.28
**HC has guideline for cervical cancer screening (*n*** **= 280)**									
Yes	58 (21)	21 (36)	37 (64)	1			1		
No	222 (79)	75 (34)	147 (66)	1.11	0.61–2.03	0.73	1.34	0.64–2.77	0.44
**HC has HPV vaccination guideline (*n*** **= 275)**									
Yes	204 (74)	67 (33)	137 (67)	1			1		
No	71 (26)	29 (41)	42 (59)	0.71	0.41–1.24	0.22	0.68	0.37–1.26	0.22

*COR* Crude Odds Ratio, *AOR* Adjusted Odds Ratio. Adjusted for each demographic characteristic. Bolded figures indicate statistically significant variables at *p* ≤ 0.05. *HC* Health centre. We used Hosmer-Lemeshow goodness-of-fit test to check if our final model had fitted the data perfectly well and the *p*-value = 0.85 from the Hosmer-Lemeshow’s goodness-of-fit test is an indicator that our model fitted the data well. The few missing data on demographic characteristics were not dropped from the analysis since the missing rates were less than 5% and were considered inconsequential based on Schafer (1999)

## Discussion

This study provides information on knowledge, attitudes, and practice of cervical cancer prevention among health workers from rural HCs in eight districts in Uganda. Health workers from rural HCs play a significant role in reaching a wider community with information regarding cervical cancer prevention measures. Though studies on knowledge, attitude and practice of cervical cancer prevention among health workers have been conducted and discussed earlier in Uganda [8] and other SSA countries [13, 53–58, 61, 62], this study is the first comprehensive one involving health workers who are based in rural HCs. This study provides valuable information which is useful in formulating comprehensive cervical cancer prevention strategies in Uganda and other SSA countries.

In our study we found a higher proportion of participants with adequate knowledge about cervical cancer compared to a study conducted in Cote d’Ivoire (55.7%) [53]. The participants in the study by Tchounga et al included student midwives [53] and this could have impacted on the level of knowledge score compared to our study in which participants were all qualified and employed.

However, knowledge was lower in our study compared to studies conducted in Ethiopia (86.9%) [54], India (85%) [63], Nigeria (98.6%) [58], Pakistan (88.1%) [64] and Burundi (76.3%) [55]. Unlike our study in which participants had to score at least 18 or more out of 25, the study conducted in India categorised participants as having good knowledge if participants were able to provide any three correct known risk factors for cervical cancer [63]. The studies conducted in Ethiopia [54], Nigeria [58] and Pakistan [64] all used scores of 50% or more to categorise participants as having good knowledge. The study conducted in Burundi had participants who were general medical practitioners [55] and are expected to have better knowledge due to their training on cervical cancer than the participants in our study who had lower levels of professional training. Therefore, there is a need to standardise scoring for adequate knowledge for cervical cancer among health workers to ensure that knowledge can be measured uniformly across countries. Lowering the cut-off value for knowledge to 50% as indicated in most of these studies would result into many health workers being categorised as having adequate knowledge. This could result in undesirable consequences as most of these health workers will not have adequate information to provide to audience. This is because health workers play a major role in communicating health behaviour to population which would require them to be well informed to pass accurate information about cervical cancer to the audience.

Most of our participants knew that early screening for cervical cancer is important in the prevention of cervical cancer. Yet, only 46 (16%) knew the correct target age group for cervical cancer screening using VIA that is recommended by Uganda Ministry of Health [7]. Furthermore, we found that some participants had misconceptions about cervical cancer risk factors, signs, and symptoms such as infection with HSV-2, having a family history of cervical cancer, over consumption of alcohol, abdominal pain, having a headache and having night sweats. The lack of knowledge on the age group recommended for cervical cancer screening using VIA, and the misconceptions exhibited by some health workers on the risk factors, signs, and symptoms of cervical cancer, need to be addressed as it is likely to have negative effects for any future preventive programmes. This is because health workers such as nurses, midwives, and clinical officers form most of the health workforce in Uganda and interface more frequently with patients and the community. Their accurate understanding of the age group recommended for cervical cancer screening, risk factors, signs, and symptoms is essential for providing correct health education messages to the patients and the community they serve.

Our study demonstrates that participants who were not trained to conduct cervical cancer screening were 61% more likely to have inadequate knowledge about cervical cancer than those who were trained. Studies conducted among health workers reported that attending training for cervical cancer prevention and treatment was significantly associated with an increase in knowledge [53, 65]. Unlike the study conducted in Tanzania which showed that younger nurses were more likely to have adequate knowledge about cervical cancer than older nurses [13], our study found no association between age of healthcare workers and their knowledge about cervical cancer. This could be because our study was conducted well after the Ugandan Ministry of Health had launched an HPV vaccination campaign in 2015 [5] and many of our participants were already made aware of cervical cancer.

In this study it was encouraging to find that most participants had positive attitudes towards participation in cervical cancer training, and prevention activities. About 88% of participants stated that they always advised their patients to be screened for cervical cancer. This is much higher compared with the findings from a study conducted in Cote d’Ivoire (38%) [53].

Our study shows that participants who were not trained to conduct cervical cancer screening were 48% times less likely to have positive attitudes towards participation in cervical cancer training, and prevention activities than participants who indicated they were trained to conduct cervical cancer screening. Given the role health workers play in providing health education about cervical cancer, there is need to increase coverage of training for health workers about cervical cancer so that negative attitudes can be changed.

Most of the female health workers in our study had been previously screened for cervical cancer. Other studies have documented a much lower uptake of cervical cancer screening by female health workers than in our study and those ranged from 6 to 41% [8, 13, 53, 54, 56, 57, 61–63, 66–73]. Female health workers who were not screened for cervical cancer cited various reasons for not attending screening including lack of access to screening services, misbeliefs about cervical cancer and screening, fear of the results and nonchalant attitude towards screening for cervical cancer. Previous studies have reported similar observations about female health workers’ lack of undertaking cervical cancer screening [8, 57, 58, 62, 63, 68, 69, 71–74].

In our study, participants’ qualifications were not associated with knowledge of cervical cancer. However, a study conducted in Cameroon found that cervical cancer knowledge was significantly associated with health workers’ qualifications [70]. This difference could be because, in the Cameroonian study, other participants such as doctors and students studying medicine, nursing and midwifery were included in that study.

### Limitations

Our study was a cross-sectional study requiring participants to recall past events and this fact could have resulted in recall bias. More women could have responded that they have ever been screened for cervical cancer due to social desirability bias. However, social desirability bias was addressed by ensuring that the questionnaire had participants’ number but no name. This gave confidence to the participants when completing the questionnaire as they were aware the information was anonymous. Participants were reassured that the information provided would be kept confidential and were encouraged to be as honest as possible while filling in the questionnaire. The study findings may not be generalised to all the nurses, midwives, and clinical officers in Uganda since the sample size was modest at 286. Furthermore, the findings may not be generalised to nurses, midwives and clinical officers who are working in referral hospitals or in private health facilities in Northern Uganda as their employers could have trained them and provided them with Strategic Plan for Cervical Cancer Prevention and Control in Uganda.

### Strength of the study

This study is the first to examine health workers’ knowledge, attitudes, and practice of cervical cancer prevention among rural health workers in Northern Uganda. The questionnaires were answered in the presence of the PI or a research assistant and this prevented health workers from discussing answers among themselves or searching information online.

## Conclusion

Our study demonstrates that there were negative attitudes and misconceptions about cervical cancer risk factors, signs, and symptoms among rural health workers as result of lack of knowledge about cervical cancer.

As part of a comprehensive strategy to achieve Sustainable Development Goal (SDG) target 3.4 which aims to reduce premature death by one third by 2030 from non-communicable diseases [23], SSA governments must develop training programme of rural health workers to improve their knowledge, attitudes, and practical skills in cervical cancer screening and treatment of precancerous lesions. Furthermore, training health workers to improve their practical skills in conducting cervical cancer screening and treatment of precancerous lesions will ensure that health workers are able to provide these services to women as part of the WHO triple-intervention by 2030 and beyond [23]. Uganda and other low-resource countries need to develop and disseminate cervical cancer prevention guidelines and health education materials which can be used by rural health workers to ensure standardise quality of services provided by rural health workers.

## Supplementary Information

**Additional file 1.** Study questionnaire

## Data Availability

The datasets generated and/or analysed during the current study are available in the Zenodo repository: 10.5281/zenodo.3722791

## Abbreviations

AOR: Adjusted odds ratio
CI: Confidence interval
CO: Clinical Officer
COR: Crude odds ratio
df: Degree of freedom
DLG: District local government
HC: Health centre
HIV/AIDS: Human immunodeficiency virus/Acquired immunodeficiency syndrome
HPV: Human papillomavirus
NGOs: Non-governmental organizations
OR: Odds ratio
Pap: Papanicolaou
PI: Principal investigator
TASO: The AIDS Support Organization
SSA: Sub-Sahara African
VIA: Visual inspection with acetic acid
WHO: World Health Organization
