# Supplementary material for: Knowledge, attitudes, and practice of cervical cancer prevention among health workers in rural health centres of Northern Uganda
Source: BMC Cancer. 2021 Feb 3;21:110. doi: 10.1186/s12885-021-07847-z (PMC7860193; doi:10.1186/s12885-021-07847-z)
Supplement: Supplementary file 1 — Additional file 1. Study questionnaire [file 12885_2021_7847_MOESM1_ESM.docx]

**Study questionnaire**

**Knowledge, attitudes, and practice of cervical cancer prevention among health workers in rural health centres of Northern Uganda**

**Participant Code: ____/____/____/** Health facility Number: ____/____/

Health facility name…………………………………Parish name……………………

Sub-county name……………………………...District name…………………..................

Date: ____/­­____/______ Interviewer’s Name……………………………

***Please mark a tick in the box applicable to your most appropriate response unless prompted for multiple responses.***

**SECTION 1:** Questions on Socio-demographic and health centre characteristics (*One alternative*)

| **S/No** | **Questions** | **Responses** |
| --- | --- | --- |
| 1.1 | Health centre level | Health centre IV 1 1  Health centre III 2 |
| 1.2 | How old are you now? | Completed Years |
| 1.3 | Gender | Male 1  Female 2 |
| 1.4 | What is your current qualification? | Enrolled Nurse 1  Registered Nurse 2  Enrolled Midwife 3  Registered Midwife 4  Enrolled Comprehensive Nurse 5  Registered Comprehensive Nurse 6  Registered Bachelor of Science Midwifery 7  Registered Bachelor of Science Nursing 8  Registered Public Health Nurse 9  Clinical Officer 10 |
| 1.5 | How many years have you been working? | Completed Years |
| 1.6 | Have you ever been trained on how to conduct screening for cervical cancer? | Yes 1  No 2 |
| 1.7 | Does the health facility have guideline for HPV vaccination? | Yes 1  No 2 |
| 1.8 | Does the health centre have any guideline for cervical cancer screening? | Yes 1  No 2 |
| 1.9 | Does the health facility receive fund for cervical cancer activities? | Yes 1  No 2 |
| 1.10 | Does this health facility have health education material about cervical cancer? | Yes 1  No 2 |
| 1.11 | Do you conduct outreach health education in the community for cervical cancer? | Yes 1  No 2 |
| 1.12 | Which period does the Strategic Plan for Cervical Cancer Prevention and Control in Uganda covers? | 2010 – 2014 1  2015 – 2020 2  2016 – 2021 3  2017 – 2022 4 |

**SECTION 2:** Hearing about cervical cancer and sources of information

| **S/No** | **Questions** | **Responses** |  |
| --- | --- | --- | --- |
| 2.1 | Have you heard of cervical cancer? | Yes 1  No 2 |  |
| 2.2 | When did you hear about cervical cancer for the first time from? | Before going for training 1  During training for current job 2  From colleague at workplace 3  During in-service training 4  Radio 5  TV 6  Others, specify……………………. 7 |  |
| **SECTION 3:** Cervical cancer screening uptake and the reasons for not screening for cervical cancer. | | | |
| **S/No** | **Questions** | **Responses** | |
| 3.1 | Have you ever been screened for cervical cancer? **(Female participants)** | Yes 1  No 2 | |
| 3.2 | If she has not been screened, probe why have she has not been screened for cervical cancer?  **(Female participants)**  ***(Multiple responses allowed)*** | Not susceptible 1  Not interested in being screen 2  Rusty/dirty equipment 3  Fear result 4  Not in age bracket for screening 5  Not at risk 6  No symptom 7  Fear male health workers  from seeing my private part 8  It’s painful 9  No one to conduct screening  in this facility 10  I don’t know health centre conducting  cervical cancer 11  Others, specify………………………. 12 | |
| 3.3 | Have you ever encouraged your partner/wife to screen for cervical cancer? **(Male participants only)** | Yes 1  No 2 | |
| 3.4 | If his response was no in 4.25, probe why he has not encouraged his wife to screened for cervical cancer?  **(Male participants)**  ***(Multiple responses allowed)*** | Not susceptible 1  I’m not interested 2  Rusty/dirty equipment 3  I fear she might find other things 4  Not in age bracket for screening 5  Not at risk 6  No symptom 7  Fear male health workers  from seeing her private part 8  It’s painful 9  No one to conduct screening  in this facility 10  I don’t know health centre screening  women for cervical cancer 11  Others, specify……………………… 12 | |

**SECTION 4: Knowledge**

Knowledge on age group for cervical cancer screening and HPV vaccination

| **S/No** | **Questions** | **Responses** |
| --- | --- | --- |
| 4.1 | What is the recommended age group for cervical cancer screening using VIA by Ministry of Health in Uganda? | 18 – 25 years 1  18 – 45 years 2  25 – 49 years 3  25 – 60 years 4 |
| 4.2 | What is the recommended age group for HPV vaccination by Uganda Ministry of Health? | 2 – 5 years 1  3 – 8 years 2  10 – 14 years 3  10 – 20 years 4 |

Which of the followings are correct risk factors for cervical cancer? Tick in the box to indicate your most accurate response.

| **S/No** | **Risk factors** | **Yes** | **No** |
| --- | --- | --- | --- |
| 4.3 | Infection with HPV |  |  |
| 4.4 | Smoking cigarette |  |  |
| 4.5 | Infection with HIV |  |  |
| 4.6 | Having multiple deliveries |  |  |
| 4.7 | Early age at first delivery |  |  |
| 4.8 | Early age at sexual debut |  |  |
| 4.9 | Long-term use of oral contraceptives |  |  |
| 4.10 | Infection with Chlamydia trachomatis |  |  |
| 4.11 | Infection with herpes simplex virus |  |  |
| 4.12 | Having multiple sexual partners |  |  |
| 4.13 | Over consumption of Alcohol |  |  |
| 4.14 | Having a family history of cervical cancer |  |  |
| Total Score | |  |  |

Which of the followings are the correct signs and symptoms of cervical cancer? Tick in the box to indicate your most accurate response.

| **S/No** | **Factors** | **Yes** | **No** |
| --- | --- | --- | --- |
| 4.15 | Foul-smelling vaginal discharge/smell |  |  |
| 4.16 | Bleeding after menopause |  |  |
| 4.17 | Bleeding after sexual intercourse |  |  |
| 4.18 | Abnormal vaginal bleeding between period |  |  |
| 4.19 | Abdominal pain |  |  |
| 4.20 | Unexplained weight loss |  |  |
| 4.21 | Having headache |  |  |
| 4.22 | Having a lot of night sweat |  |  |
| Total score | |  |  |

Which of the followings are the correct methods of cervical cancer prevention? Tick in the box to indicate your most accurate response.

| **S/No** | **Methods** | **Yes** | **No** |
| --- | --- | --- | --- |
| 4.23 | Vaccination against HPV |  |  |
| 4.24 | Using condom during sexual encounter |  |  |
| 4.25 | Avoiding multiple sexual partner |  |  |
| 4.26 | Delaying initiation of sex after 18 years |  |  |
| 4.27 | Male circumcision |  |  |
| 4.28 | Avoiding prolong use of oral contraceptive pills |  |  |
| 4.29 | Avoiding smoking cigarette |  |  |
| 4.30 | Avoiding multiple delivery/births |  |  |
| 4.31 | Early screening |  |  |
| Total score | |  |  |

**SECTION 5: Attitudes**

The following statements below is about your attitudes toward cervical cancer. Use these ratings to indicate your most likely attitudes by making a tick in the appropriate boxes.

SD=Strongly Disagree, D=Disagree, N=Neutral, A=Agree, SA=Strongly Agree.

| **S/No** | **Statements** | **SD (1)** | **D (2)** | **N (3)** | **A (4)** | **SA (5)** | |
| --- | --- | --- | --- | --- | --- | --- | --- |
| 5.1 | I always advise my patients to screen for cervical cancer. |  |  |  |  |  | |
| 5.2 | I discuss cervical cancer in our staff meeting. |  |  |  |  |  | |
| 5.3 | I will be happy to see that my children/sister are  immunised against HPV. |  |  |  |  |  | |
| 5.4 | Cervical cancer is not a serious health problem, so screening is just a burden. |  |  |  |  |  | |
| 5.5 | Even if we screen and find a woman with  precancerous lesion, there is nothing we can do. |  |  |  |  |  | |
| 5.6 | I don’t think it is necessary to screen for cervical cancer in our health facility |  |  |  |  | |  |
| 5.7 | Government has not shown commitment about cervical cancer so why bother us. |  |  |  |  | |  |
| 5.8 | I am not interested in cervical cancer prevention because partners/NGOs do their work without developing our capacity to implement cervical cancer control program on our own. |  |  |  |  | |  |
| 5.9 | I will participate in cervical cancer prevention  program if I am going to be paid money cash. |  |  |  |  | |  |
| 5.10 | I am willing to participate in a training for cervical cancer prevention if organised by Gulu University & University of New South Wales – Australia. |  |  |  |  | |  |
| 5.11 | I am willing to participate in a training for cervical cancer prevention if organised by Ministry of Health Uganda |  |  |  |  | |  |
| 5.12 | I am willing to participate in a training for cervical cancer prevention if organised by NGOs |  |  |  |  | |  |
| 5.13 | I am more like to screen women for cervical cancer if I am trained and given equipment & consumables |  |  |  |  | |  |

**Result of Questionnaire:** 1**.** Completed 2. Refused 3. Partly Completed

**Supervisor’s Verification:** 1.Yes 2. No
